# Knowledge and attitude toward transcranial magnetic stimulation among rehabilitation specialists in Saudi Arabia

**DOI:** 10.3389/fbioe.2024.1352170

**Published:** 2024-03-18

**Authors:** Alaa M. Albishi, Saja A. Alhadlaq, Rahaf T. Altowairqi, Mohammed F. Alharbi, Abdulrahman M. Alsubiheen, Manal H. Alosaimi, Shahid Bashir, Ahmad O. Alokaily

**Affiliations:** ^1^ Department of Health Rehabilitation Sciences, College of Applied Medical Sciences, King Saud University, Riyadh, Saudi Arabia; ^2^ Department of Speech-Language Pathology and Audiology, College of Medical Rehabilitation Sciences, Taibah University, Madina, Saudi Arabia; ^3^ Radiological Sciences Department, College of Applied Medical Sciences, King Saud University, Riyadh, Saudi Arabia; ^4^ Neurosciences Center, King Fahad Specialist Hospital–Dammam, Dammam, Saudi Arabia; ^5^ Department of Biomedical Technology, College of Applied Medical Sciences, King Saud University, Riyadh, Saudi Arabia; ^6^ King Salman Center for Disability Research, Riyadh, Saudi Arabia

**Keywords:** rehabilitation, knowledge, attitude, physical therapist, brain stimulation, transcranial magnetic stimulation (TMS), clinicians, Saudi Arabia

## Abstract

Research has demonstrated the benefits of transcranial magnetic stimulation (TMS) in rehabilitation. TMS has been widely used in clinical and research settings for individuals with and without neurological dysfunctions. Therefore, understanding the knowledge and attitudes of rehabilitation specialists regarding TMS is crucial for its application. To our knowledge, no such studies have previously been conducted in the rehabilitation field. Therefore, this study is the first to assess rehabilitation specialists’ knowledge of and attitudes toward TMS. An observational cross-sectional study using a self-administered online survey was conducted among 102 rehabilitation specialists to assess their knowledge and attitudes regarding TMS application in rehabilitation sciences. Descriptive and inferential statistics were used to describe the knowledge and attitudes of rehabilitation specialists toward TMS and examine the impact of different factors such as gender, education level, acceptability, and practice on these outcomes. Rehabilitation specialists who participated in this study showed a limited level of general knowledge of TMS in rehabilitation (7.81 ± 6.20, 37.19%). However, a significant association between educational levels and knowledge was found. Higher knowledge scores were observed for specialists with post-graduate degrees compared to those with only a bachelor’s degree. Moreover, knowledge level, experience, and availability of TMS equipment in the workplace led to a positive attitude toward TMS among rehabilitation specialists. A low knowledge level among rehabilitation specialists was attributed to their level of education. Nevertheless, specialists showed an overall positive attitude toward TMS. Therefore, customized medical education is necessary to incorporate TMS theory and applications into neuroscience and rehabilitation courses for rehabilitation specialists as it holds significant promise as a therapeutic tool.

## 1 Introduction

Transcranial magnetic stimulation (TMS) is a noninvasive brain stimulation (NIBS) technique that can directly manipulate neural activity in different cortical areas (Rossini and Rossi, 2007). It generates a magnetic field on a coil resting on the scalp, which induces electric currents in the brain, spinal roots, or nerves (Londero et al., 2006; Chail et al., 2018). This electrical current can depolarize neurons or their axons if the current’s amplitude, duration, and direction are appropriate (Chail et al., 2018); as a result, increased or decreased cortical activity occurs in that area of the brain, which can be related to functional changes (Londero et al., 2006; Lefaucheur et al., 2014). TMS can be used in clinical and research settings for individuals with and without neurological dysfunctions (US Food and Drug Administration, 2015; Saudi Food and Drug Authority SFDA, 2016; AlHadi et al., 2017; Deng et al., 2020; Kim et al., 2011; Zeina et al., 2014; Baradi and Shahid, 2017; Al-Sultan et al., 2019). The United States Food and Drug Administration (FDA) and the Saudi FDA have approved the clinical use of TMS for treating adult patients with depression who have failed to show significant improvement with antidepressant medications (U.S. Food and Drug Administration, 2015; Saudi Food and Drug Authority SFDA, 2016). In addition to its medical use, TMS has been widely applied in different rehabilitation settings for individuals with neurological and musculoskeletal conditions (Kim et al., 2011; Zeina et al., 2014; AlHadi et al., 2017; Baradi and Shahid, 2017; Al-Sultan et al., 2019; Deng et al., 2020). Specifically, stimulating the motor cortex with TMS improves performance among patients with motor impairments (Zeina et al., 2014; Baradi and Shahid, 2017).

Enhancing motor performance is a core goal for physical therapy interventions; therefore, in the past few decades, there has been vigorous growth in the range of rehabilitation technologies, such as noninvasive brain stimulation (NIBS), that can improve the recovery of patients with motor impairments (Winstein et al., 2016; Palmer et al., 2018; Winstein and Varghese, 2018; Al-Hussain et al., 2021; Bashir et al., 2021; Braun and Wittenberg, 2021). Specifically, combining TMS with different rehabilitation techniques to improve motor recovery has been widely investigated. For instance, TMS with constrained induced movement therapy (CIMT) has been shown to be superior to CIMT alone for improving upper limb functions in patients who have suffered a stroke (Abdullahi et al., 2023). In addition, TMS applications have been associated with improved motor function, learning, and consolidation in healthy individuals and individuals who have suffered a stroke (Dayan and Cohen, 2011). A recent study demonstrated the efficacy and feasibility of utilizing TMS in different rehabilitation settings (Braun and Wittenberg, 2021). TMS can also be used to investigate changes in motor cortex excitability and muscle representation in the motor cortex following different rehabilitation paradigms (Palmer et al., 2018; Braun and Wittenberg, 2021; Abdullahi et al., 2023). For example, increased cortical excitability and upper limb muscle representation in the primary motor area (M1) have been reported following rehabilitation intervention for patients who have suffered a stroke (Braun and Wittenberg, 2021). Thus, incorporating TMS into rehabilitation is important for augmenting patients’ motor recovery and facilitating functional independence.

However, the effectiveness of TMS could vary depending on the coli shape, size, orientation, and magnetic field intensity, frequency, and duration (Rossini and Rossi, 2007; Rossi et al., 2009; León Ruiz et al., 2018). The absence of a standardized protocol for TMS modalities has limited achieving optimal and effective therapeutic outcomes (Kim et al., 2020; Lefaucheur et al., 2020; Vallejo et al., 2023). In addition, individual variability in the degree to which there is facilitation or inhibition has been reported (Maeda et al., 2000). The variability in the outcomes of TMS therapy among patients may be attributed to factors such as genetic variations, the timing of TMS application in regard to disease severity, the underlying pathology, and the specific parameters employed during the therapy administration (Maeda et al., 2000; Chervyakov et al., 2015; Vallejo et al., 2023). In fact, there is still a need for more evidence regarding the reliability of TMS effects within and among studies in research and clinical settings (Nazarova and Asmolova, 2022).

Although some current evidence supports the use of TMS in medical and research settings, its usability in research and clinical studies in Saudi Arabia has not been explored in depth. This could be related to different factors. One of the most critical factors reported in previous studies is patients’ misconceptions about their examination or treatment, as explained by their healthcare provider; this, in turn, is directly impacted by the healthcare provider’s knowledge (Dowman et al., 2005). Current studies have shown that knowledge of and positive attitudes toward brain stimulation techniques significantly correlate with therapeutic referrals (Dauenhauer et al., 2011; AlHadi et al., 2017; Deng et al., 2020). A study conducted in Saudi Arabia involving 96 psychiatrists assessed their knowledge and acceptability of repetitive TMS (rTMS) utilizing an online survey. The study showed that 80% of respondents thought they had good knowledge of rTMS, 79% had a positive attitude toward this technique, and more than half were willing to consider it as a treatment approach (AlHadi et al., 2017). The survey concluded that knowledge and attitudes were correlated with the implementation of rTMS among psychiatrists in Saudi Arabia. These findings aligned with similar studies in other countries (Gazdag et al., 2004; Dan et al., 2014; Raharjanti et al., 2022). Thus, knowledge and attitudes are important factors to consider in expanding and facilitating the use of TMS in rehabilitation.

While numerous studies have explored the knowledge and attitudes of patients and psychiatrists towards transcranial magnetic stimulation (TMS) (Gazdag et al., 2004; Dowman et al., 2005; Dauenhauer et al., 2011; AlHadi et al., 2017; Deng et al., 2020), there has been limited investigation into the understanding and perceptions of TMS among professionals outside the field of psychiatry. The significance of comprehending the rehabilitative aspects and practicality of TMS cannot be overstated, as it directly impacts its clinical implementation. Notably, no previous studies have examined the knowledge and attitudes of rehabilitation specialists towards TMS. Hence, this study aims to conduct the first assessment of TMS knowledge and attitudes among rehabilitation specialists in Saudi Arabia.

## 2 Materials and methods

An observational cross-sectional study was carried out through the anonymous distribution of an online survey. A self-administered questionnaire on knowledge of and attitude toward TMS was adapted from a previous study that evaluated this topic among psychiatrists in Saudi Arabia (AlHadi et al., 2017). The questionnaire was modified to measure knowledge and attitudes regarding the applications of TMS in rehabilitation. A convenience sampling approach targeted registered rehabilitation specialists such as physical, Occupational, and speech-language therapists in Saudi Arabia to explore their knowledge and attitudes regarding TMS applications in the rehabilitation sciences.

### 2.1 Sample size calculation

The sample size for this study was determined using a calculation formula commonly used for cross-sectional studies (Pourhoseingholi et al., 2013):

$$n=\frac{Z^{2}\times P\left(1-P\right)}{d^{2}}$$

By considering a 95% confidence level (Z = 1.96), an expected prevalence (P) of 0.60, and a precision (d) of 0.10, similar to a previous study (AlHadi et al., 2017). A required sample size of 93 participants was determined. The questionnaire was distributed electronically to approximately 250 registered rehabilitation therapists, and those who responded were included in the study, resulting in a response rate of 37.20%.

### 2.2 Data collection

The survey on knowledge and attitudes regarding TMS applications in rehabilitation was distributed via the Internet to registered Rehabilitation specialists due to the available access to their database in universities and colleges, general and private hospitals, and research institutions. Licensed rehabilitation therapists who practice in Saudi Arabia (n = 102) were included in this study. All participants were recruited and provided informed consent after approval of the study by the Internal Review Board of King Saud University (no. E-22-7271).

### 2.3 Questionnaire validation

The modified questionnaire consisted of 43 questions grouped into three main sections: demographics (12 questions), knowledge (21 questions), and attitudes (10 questions). The demographic items were used to collect information regarding each participant’s age, gender, region, level of education, subspecialty, type of workplace, number of attended conferences per year, and preferred source of information. Moreover, each participant was asked if they had sufficient knowledge of TMS applications, underwent training abroad in the past 6 months, or had any experience with TMS. The knowledge section measured general knowledge of TMS applications, practices, and contraindications to TMS in rehabilitation sciences, whereas the attitude-related questions assessed the practitioner’s perceptions of TMS.

Furthermore, six independent experts from the Department of Rehabilitation Sciences and the Department of Biomedical Technology at King Saud University were selected to assess the validity of the questionnaire content based on their seniority level and research experience. The research objectives, domains (knowledge and attitude), and the representing items were distributed with clear instructions to evaluate each item based on clarity and relevance to the tested domain. Experts were asked to critically assess and judge the degree of relevance and clarity by rating each item on a scale of 1–4, with “1″being irrelevant/unclear and “4″being highly relevant/clear. Recommendations from experts were incorporated to enhance the questionnaire’s face validity and item clarity.

To evaluate the content validity of the modified survey, the expert’s item scores were converted to 1 (if the item relevance score was 3 or 4) or 0 (if the item was given a relevance score of 2 or 1). The scale-level content validity index was computed based on the average method (S-CVI/Avg) to assess the scale’s content validity (Yusoff, 2019). To reach satisfactory content validity, achieving an S-CVI/Avg score of at least 0.83 is necessary (Yusoff, 2019). The modified questionnaire attained satisfactory levels of content validity of 0.96 and 0.93 for the knowledge and attitude domains, respectively.

### 2.4 Data analysis

Statistical analyses were performed with IBM’s SPSS software version 29. The scoring system was adapted and modified from (AlHadi et al., 2017) and applied to evaluate the participants’ knowledge and attitudes regarding TMS. For the knowledge assessment, one point was given for each correct response and zero for each incorrect or uncertain response. For knowledge, a continuous variable with a maximum possible score of 21 points was computed by summing the attained per-question score for each respondent. The knowledge score of a respondent was categorized based on ranges developed from the achieved scores into low (0–10), moderate (11–16), and high (15,17–20).

Further, the attitude section used a 5-point Likert scale with the possibilities of strongly agree, agree, neutral, disagree, and strongly disagree. The responses were scored from 1–5 points, with a maximum score of 50 points for the attitude section. The scoring order for negative statements was reversed. The attitude score was classified as negative for an attained score of 0–24 points and positive for an attuited score of 25–50. Negative statements in the questionnaire were items 1–4, and positive statements were items 5–10.

Descriptive statistics, including frequency, mean, and standard deviation (SD) were used to demonstrate the participants’ characteristics, performance, and perceptions. Additionally, the computed continuous variables of knowledge and attitude scores were evaluated using the Shapiro–Wilk normality test. Nonparametric tests were used due to the skewed distributions of the computed scores (Shapiro–Wilk, *p* < .05). The Kruskal–Wallis, Mann–Whitney U, and Spearman’s rank correlation tests were used to examine the effect of different factors on the knowledge of and attitudes toward TMS among rehabilitation specialists. The Kruskal–Wallis was used with factors of more than two levels, such as education level (bachelor, master, or doctorate degree), subspeciality (general, neuro, or orthopedic rehabilitation), number of annually attended conferences, region, main place of practice, and source of knowledge followed by *post hoc* analysis via Mann-Whitney U to investigate significant effects on knowledge and attitude scores. Direct Mann-Whiteny U was directly applied to investigate the significant effects of two-level factors such as gender, TMS accessibility, TMS experience, and abroad training. Spearman’s rank correlation test assessed the association between rehabilitation specialist’s age and attained knowledge and attitude scores. All data were presented as mean ± SD. The study results were considered significant at *p* < .05.

## 3 Results

### 3.1 Participant characteristics

A total of 102 rehabilitation specialists completed the study questionnaire. The participants were 25–55 years old, with an average age of 36.31 ± 7.21 years, and the majority were female (57, 55.88%). Rehabilitation specialists with bachelor’s degrees in rehabilitation sciences represented 45.10% of the sample, followed by senior specialists with a master’s degree (29.41%), and doctoral degree holders amounted to 25.49%. The participants practiced under several rehabilitation subspecialties, with a majority in general rehabilitation (73.53%) and neural rehabilitation (19.61%). The remaining responders practiced in orthopedics rehabilitation.

The participants were from all regions of Saudi Arabia. The majority were in the central region (69.61%). The participation rate for the other regions was 8.82% for the western and eastern regions, 5.88% for the northern region, and 6.86% for the southern region. Approximately 69% of respondents indicated mainly utilizing scientific articles to keep their knowledge current. Most respondents did not have TMS experience (81.37%) and reported insufficient knowledge of TMS applications. The demographic information is presented in Table 1.

**TABLE 1:** Demographic factors.

Variable	Subcategory	*n* (%)
Gender	Female	57 (55.88)
	Male	45 (44.12)
Education	Doctoral	26 (25.49)
	Master	30 (29.41)
	Bachelor	46 (45.10)
Subspecialty	General rehabilitation	75 (73.53)
	Neurorehabilitation	20 (19.61)
	Orthopedics rehabilitation	7 (6.86)
Number of conferences attended annually	1	47 (46.08)
	2–3	41 (40.20)
	More than 3	14 (13.73)
Region	Central	71 (69.61)
	Western	9 (8.82)
	Eastern	9 (8.82)
	Northern	6 (5.88)
	Southern	7 (6.86)
Main place of practice	General hospital	37 (37.25)
	Teaching hospital	3 (43.14)
	Private practice	16 (15.69)
	Rehabilitation center	4 (3.92)
	University	42 (41.18)
The main source of knowledge	Articles	70 (68.63)
	Conferences	8 (7.84)
	Textbooks	9 (8.82)
	Discussions with colleagues	15 (14.71)
Have you had training abroad for more than 6 months?	Yes	29 (28.43)
	No	72 (70.59)
Do you have a TMS machine at your workplace?	Yes	10 (9.80)
	No	92 (90.20)
Do you have any previous experience with a TMS machine?	Yes	19 (18.63)
	No	83 (81.37)
Do you have sufficient knowledge about TMS applications?	Yes	22 (21.57)
	No	80 (78.43)

### 3.2 Assessment of TMS knowledge

The knowledge-related items and response rates are presented in Table 2. Most of the participants indicated that they were aware that TMS has the potential to be used as a treatment tool for neurological diseases (58.82%). However, only 30.39% of respondents indicated that TMS could be a diagnostic tool for neurological diseases. Moreover, approximately 64% indicated that TMS can activate various specific areas in the brain. In contrast, the knowledge assessment showed lower knowledge of TMS contraindications, such as a history of epilepsy, pacemakers, and implanted devices, as well as the unnecessary requirement for hospital admission or muscle relaxants. Overall, the average knowledge score was 7.81 ± 6.20 SD. Only 40% of the respondents obtained more than 50% of the maximum possible knowledge score. Moreover, the knowledge scores indicated that 61% of participants had low knowledge levels, 30% had moderate knowledge levels, and only 9% had high knowledge levels.

**TABLE 2:** Knowledge of TMS among rehabilitation specialists.

Questions	Frequency (%) of responders		Mean ± SD
	Correct	Incorrect	
1- TMS is used as a treatment tool for neurological patients. (T)	60 (58.82)	42 (41.18)	0.59 ± 0.49
2- TMS is used as a diagnostic tool for neurological patients. (T)	31 (30.39)	71 (69.61)	0.30 ± 0.46
3- TMS therapy requires hospital admission. (F)	35 (34.31)	67 (67.69)	0.34 ± 0.48
4- TMS is used in psychiatric hospitals only. (F)	46 (45.10)	56 (54.90)	0.45 ± 0.50
5- TMS can be administered only under general anesthesia. (F)	48 (30.39)	54 (30.39)	0.47 ± 0.50
6-TMS can cause permanent brain damage. (F)	46 (45.10)	56 (54.90)	0.45 ± 0.50
7- TMS can stimulate specific areas of the brain. (T)	64 (62.75)	38 (37.25)	0.63 ± 0.49
8- TMS is an outdated therapy. (F)	37 (36.27)	65 (63.73)	0.36 ± 0.48
9- TMS causes moderate to severe pain. (F)	35 (34.31)	67 (65.69)	0.34 ± 0.48
10- TMS is an FDA-approved treatment method for stroke patients. (F)	4 (3.92)	98 (96.08)	0.04 ± 0.20
11- TMS has shown significant results in treating some neurological patients.	46 (45.10)	56 (54.90)	0.45 ± 0.50
12- TMS is contraindicated in patients with intracranially implanted metallic objects. (T)	46 (45.10)	56 (54.90)	0.45 ± 0.50
13- TMS is contraindicated in patients with epilepsy/seizures. (T)	35 (34.31)	67 (67.69)	0.34 ± 0.48
14- TMS is contraindicated in patients with a pacemaker. (T)	41 (40.20)	61 (59.80)	0.40 ± 0.49
15- TMS is a contraindication in pregnancy. (T)	35 (34.31)	67 (65.69)	0.36 ± 0.48
16- TMS is contraindicated in patients with dental fillings. (F)	20 (19.61)	82 (80.39)	0.20 ± 0.40
17- TMS is used more often in Saudi Arabia compared to other countries. (F)	30 (29.41)	72 (70.59)	0.29 ± 0.46
18- TMS can be used to stimulate the motor cortex. (T)	45 (44.12)	57 (55.88)	0.44 ± 0.50
19- TMS can be performed without a muscle relaxant. (T)	23 (22.55)	79 (77.45)	0.23 ± 0.42
20- TMS can be used over the age of 65. (T)	23 (22.55)	79 (77.45)	0.23 ± 0.42
21- The recommended number of TMS sessions varies according to the patient’s condition. (T)	45 (44.12)	57 (55.88)	0.44 ± 0.52
Knowledge Score			7.81 ± 6.20

The mean attained scores (and SDs) were 5.63 ± 0.80, 9.10 ± 1.15, and 10.19 ± 1.23 for those holding bachelor’s, master’s, and doctoral degrees, respectively. The knowledge scores were compared across levels of education (bachelor’s, master’s, and doctoral) to examine their effect on TMS knowledge. The knowledge score significantly differed across education levels (Kruskal–Wallis test: *p* = .003). Post-hoc analysis showed that specialists with post-graduate education had significantly higher TMS knowledge compared with specialists with bachelor’s degrees (doctoral’s vs. bachelor’s, Mann–Whitney U test: Z = −3.11, *p* < .002) (master’s vs. bachelor’s, Mann–Whitney U test: Z = −2.39, *p* < .017). In addition, specialists who indicated previous experience with TMS applications had significantly higher knowledge than those without experience (Mann–Whitney U test: Z = −2.99, *p* < .003).

Statistical investigation of other factors such as gender, region, main source of knowledge, type of workplace, subspecialty, training abroad, accessibility to TMS, or claiming sufficient knowledge of TMS application did not reveal a significant effect on TMS knowledge (*p* > .05). Moreover, the results indicated no correlation between the specialists’ knowledge and age (Spearman’s rank correlation: *p* > .05).

Additionally, further statistical analysis was carried out on the subgroup of neurorehabilitation specialists (n = 20) to evaluate the variable that might contribute to their knowledge of and attitude toward TMS. Similar to the overall respondents, neurorehabilitation specialists with previous experience with TMS attained significantly higher knowledge scores than those without experience. (Mann–Whitney U test: Z = −2.07, *p* = .04). However, education level, gender, the primary source of knowledge, and TMS accessibility had no significant effect on TMS knowledge (*p* > .05).

### 3.3 Attitudes toward TMS

The questionnaire items assessing the rehabilitation specialists’ attitudes and the response rate are shown in Table 3. A large proportion of the participants had a neutral perception of TMS in general. However, 55.88% reported that all rehabilitation specialists should attend training courses on TMS applications. Approximately 43.14% of respondents had confidence that TMS applications would enhance the quality of care in rehabilitation, and 43.14% indicated that sufficient knowledge is crucial in neurorehabilitation practice. Furthermore, 41.18% of the respondents believed that more TMS research studies should be implemented in Saudi Arabia, and only 9.8% indicated that they would not consent to participate in a TMS study if they had suffered from a brain injury. In contrast, only 25.49% would recommend TMS as a potential adjunct therapy, and 20.59% felt that TMS should only be used as a last resort. Approximately 23% agreed that there is still insufficient evidence of TMS efficacy in rehabilitation contexts. Nevertheless, the average attitude score was 33.13 ± 4.54 (66.26%) out of a maximum score of 50. A significant number of respondents showed a positive attitude toward TMS in rehabilitation. Approximately 98% of the participating specialists attained more than 50% of the possible points, and only 2% had a negative attitude (n = 2).

**TABLE 3:** Attitudes toward TMS among rehabilitation specialists.

Questions	Frequency (%) of responders					Mean ± SD
	Strongly agree	Agree	Neutral	Disagree	Strongly disagree	
1-I believe that TMS treatment is too risky.	2 (1.96)	11 (10.78)	55 (53.92)	29 (28.43)	5 (4.90)	3.24 ± 0.78
2- TMS should only be used as a final treatment option.	2 (1.96)	19 (18.63)	52 (50.98)	24 (23.53)	5 (4.90)	3.11 ± 0.83
3- TMS is typically used more often on individuals with low socioeconomic status.	1 (0.98)	8 (7.84)	61 (59.80)	22 (21.57)	10 (9.80)	3.31 ± 0.79
4- There is no sufficient evidence of the efficacy of TMS in the field of neurorehabilitation.	3 (2.94)	21 (20.59)	55 (53.92)	19 (18.63)	4 (3.92)	3.00 ± 0.82
5- I would recommend TMS as an adjunct therapy option to my patients	6 (5.88)	20 (19.61)	68 (66.67)	6 (5.88)	2 (1.96)	3.22 ± 0.72
6- Knowing TMS is essential in the practice of rehabilitation.	17 (16.67)	27 (26.47)	42 (41.18)	12 (11.76)	4 (3.92)	3.40 ± 1.02
7- I would consent to participate in TMS studies if I suffered an injury to the central nervous system.	7 (6.86)	28 (27.45)	57 (55.88)	7 (6.86)	3 (2.94)	3.28 ± 0.81
8- The applications of TMS will improve the quality of care.	5 (4.90)	39 (38.24)	54 (52.94)	4 (3.92)	0 (0)	3.44 ± 0.65
9- TMS studies should be implemented at all rehabilitation centers in Saudi Arabia	15 (14.71)	27 (26.47)	52 (50.98)	6 (5.88)	2 (1.96)	3.46 ± 0.88
10- All rehabilitation specialists should have special training courses about TMS.	24 (23.53)	33 (32.35)	33 (32.35)	11 (10.78)	1 (0.98)	3.67 ± 0.98
Attitude Score						33.13 ± 4.54

Statistical testing was performed to investigate the factors that affect perceptions of TMS in rehabilitation. The computed attitude score significantly differed across education levels (Kruskal–Wallis test: *p* = .04). Post-hoc analysis showed that specialists with master degrees were more positive toward TMS than specialists with bachelor’s degrees (bachelor’s vs. master’s, Mann–Whitney U test: Z = −2.43, *p* = .015). However, no significant differences were found between bachelor’s or master’s degree holders compared to doctoral degree holders (*p* > 0.05). Moreover, the attitude score was found to be significantly more positive in participants who reported the presence of TMS equipment in their workplace (Mann–Whitney U test: Z = −2.29, *p* = .022), had previous experience with TMS devices (Mann–Whitney U test: Z = −2.79, *p* < .005), and claimed to have sufficient knowledge about TMS applications (Mann–Whitney U test: Z = −4.13, *p* < .001). There was no effect of other factors such as gender, region, primary source of knowledge, type of workplace, subspecialty, attended conferences, and training abroad on attitudes toward TMS application (*p* > .05). No significant association between age and attitude score was found (*p* > .05). In addition, the subgroup statistical analysis on the neurorehabilitation specialists only, revealed no significant effects of education level, gender, previous TMS experience, accessibility to TMS, and training abroad on the attitude toward TMS among neurorehabilitation specialists (*p* > .05).

### 3.4 Scale reliability

The internal consistency of the assessment questionnaire was assessed using Cronbach’s alpha (α) reliability test. The reliability test results indicated adequate internal consistency, with a Cronbach’s α value of .92 for the knowledge section and .73 for the attitude section. The overall internal consistency of the questionnaire was .83, indicating a reliable measure of both knowledge and attitudes (Terwee et al., 2007).

## 4 Discussion

This study used a self-administered survey to explore rehabilitation knowledge of and attitudes toward TMS in Saudi Arabia. The number of female respondents was slightly higher than that of males, which is similar to previous studies; a higher proportion of females could reflect that rehabilitation practice in Saudi Arabia is as independent as other medical and health specialties with discrepancy of gender distribution across all the medical fields (Hussein et al., 2022). Our respondents’ mean age was similar to other studies (Mouton et al., 2014). Most of our respondents were bachelor’s degree holders practicing under various rehabilitation subspecialties, and only a few specialized in neurorehabilitation. A similar statistic was reported in previous studies, demonstrating relatively low numbers of post-professional and specialized therapists compared to the number of bachelor’s physical therapy degrees and clinical Doctor of Physical Therapy (DPTs) (Bindawas, 2014; Alghadir et al., 2015; Yorke et al., 2016). Most of our responses were from the central region of Saudi Arabia due to the large number of rehabilitation programs and a wide range of hospitals, as reported in previous studies in Saudi Arabia (Bindawas, 2014; Al-Eisa et al., 2016).

One of the study’s aims was to assess basic TMS knowledge among different subgroups of rehabilitation specialists. Most of the respondents did not have TMS equipment or experience and felt their knowledge of TMS applications was limited. The lack of TMS equipment could explain the low TMS knowledge among our participants, as previous research has demonstrated that a lack of equipment and workspace in rehabilitation is considered a common barrier to translating knowledge into practice while providing equipment would be regarded as a facilitator of knowledge applicability (Graham et al., 2006; Weatherson et al., 2017; Moncion et al., 2020). Most participants indicated that they were aware that TMS has the potential to be used as a treatment tool for neurological diseases and could be used to activate various brain areas, but not as a diagnostic tool. This could be attributed to the fact that physicians commonly handle patients’ diagnoses before their referral for rehabilitation interventions in most practices in Saudi Arabia, which could impact our participants’ knowledge of TMS usage as a diagnostic tool (Al-Eisa et al., 2016). We also found a lack of knowledge among our participants regarding TMS contraindications and the unnecessary use of hospital admission or muscle relaxants. This could be attributed to discrepancies between studies on TMS safety and what is considered an absolute TMS contraindication rather than a reason to apply it with caution (Wassermann, 1998; Tope and Shellock, 2002; Perera et al., 2016; AlHadi et al., 2017; McClintock et al., 2017; Shah et al., 2019; Deng et al., 2020; Vallejo et al., 2023).

The overall average knowledge score was low (7.81 ± 6.20), and only 40% of the respondents obtained more than 50% of the maximum possible knowledge score. This could be because most of our participants did not have TMS equipment in their workplaces and were not post-professional degree holders, which might be attributed to the lack of post-professional rehabilitation and residency programs in Saudi Arabia (Bindawas, 2014; Alghadir et al., 2015). In addition, limited TMS research has been conducted among rehabilitation specialists in Saudi Arabia (Al-Hussain et al., 2021; Bashir et al., 2021). TMS is still considered a new concept to be addressed in rehabilitation in Saudi Arabia, and brain stimulation is not covered in the curriculum of bachelor’s degree programs in rehabilitation science. We also found that the overall knowledge score was higher for respondents with master’s and doctoral degrees and demonstrated a significant relationship between TMS knowledge and education levels in all of our respondents, including the subgroup of neurorehabilitation specialists. Additionally, specialists with previous experience with TMS applications had significantly more knowledge than those without experience. This study did not find an effect of gender, age, region, main source of knowledge, type of workplace, or subspecialty on overall TMS knowledge. These findings are aligned with previous studies conducted among psychiatrists evaluating their knowledge of TMS in Saudi Arabia (Walter et al., 2001; AlHadi et al., 2017).

Overall, most of the participants had a neutral perception of TMS. However, more than half of the participants reported that all rehabilitation specialists should attend training courses on TMS applications, in which interns speak for the need for TMS training and educational courses among rehabilitation specialists. Approximately 43.14% of respondents had confidence that TMS applications would enhance the quality of care in rehabilitation and indicated that sufficient knowledge is crucial in neurorehabilitation practice. Thus, emphasizing knowledge of TMS is essential for its application by rehabilitation specialists. Only 25.49% of the respondents indicated that they would recommend TMS as a potential adjunct therapy; 23% agreed that there is still no sufficient evidence of TMS efficacy in rehabilitation, while 41.18% of participants believed that more TMS research studies should be implemented in Saudi Arabia. This reflects the current controversy and limitations of TMS applications and effectiveness in the literature (Vallejo et al., 2023), as well as specialists ' need for more education programs and the implementation of TMS in Saudi Arabia. More TMS research is needed among rehabilitation specialists in Saudi Arabia. The average attitude score was 40.58 ± 4.91 out of a maximum score of 65 (or 62.43%), which showed a significant positive attitude toward TMS in rehabilitation. The attitude score was significantly more positive in participants who reported the presence of TMS equipment in their workplace, had previous experience with TMS devices, and reported having sufficient knowledge about TMS applications. Similar positive attitudes toward TMS among psychiatrists in Saudi Arabia were reported in previous studies (Walter et al., 2001; AlHadi et al., 2017). These results and previous studies support the idea that knowledge, experience, and TMS availability are crucial for facilitating TMS implementation among rehabilitation specialists.

Based on this study’s findings, we recommend advancing knowledge on the use of TMS among rehabilitation specialists; we recommended incorporating TMS topics in educational programs, especially in the courses that address neuroplasticity concepts in rehabilitation, to better understand the mechanism and rationale for using TMS in Saudi Arabia. Also, rehabilitation students in undergraduate and graduate rehabilitation programs should be encouraged to have their graduation research projects on TMS. Moreover, since FDA approves TMS for psychiatric disorders and the TMS equipment is available in psychiatric departments, building collaboration with these departments would foster the knowledge and attitude toward using TMS in rehabilitation and increase access to TMS devices. Conducting workshops and scientific meetings on TMS applications and safety would also improve the knowledge and attitude toward TMS in rehabilitation.

Nevertheless, this study had some limitations. The survey was implemented with Google Forms, which implies that the responding specialists use computer support. Moreover, most of our participants were from the central region of Saudi Arabia, which could limit the generalizability of the results to the other areas. Additionally, the survey targeted rehabilitation specialists and did not include all healthcare providers, such as medical doctors and nurses. Further validation of our findings in other populations of medical practitioners is needed to assess their knowledge and attitudes toward TMS in rehabilitation. Another limitation of this study is that it did not focus on neurorehabilitation specialists or neurologists to capture their knowledge and attitude toward TMS application in Saudi Arabia. Our study only included a small sample of neurorehabilitation specialists. Further study on a larger sample of neurorehabilitation and neurological clinicians is needed to validate our results. Our questionnaire did not focus on different TMS modalities nor specific TMS risks, which might not help differentiate the lack of knowledge in different TMS applications and their safety. Our current study represents an essential first step toward assessing rehabilitation specialists’ general knowledge and attitudes toward TMS in Saudi Arabia.

## 5 Conclusion

Most rehabilitation specialists had insufficient knowledge of TMS, which was attributed to factors such as level of education. Therapists with higher education levels had higher knowledge scores than those with lower educational levels. A high educational level, years of experience, and availability of TMS equipment in the workplace led to a positive attitude toward TMS among rehabilitation specialists.

## Data Availability

The raw data supporting the conclusions of this article will be made available by the authors, without undue reservation.
